# High‐Efficiency Spatial‐Wave Frequency Multiplication Using Strongly Nonlinear Metasurface

**DOI:** 10.1002/advs.202101212

**Published:** 2021-07-15

**Authors:** Hai Peng Wang, Yun Bo Li, Shi Yu Wang, Jia Lin Shen, He Li, Shi Jin, Tie Jun Cui

**Affiliations:** ^1^ State Key Laboratory of Millimeter Waves Southeast University Nanjing 210096 China; ^2^ National Mobile Communications Research Laboratory Southeast University Nanjing 210096 China

**Keywords:** nonlinear active metasurfaces, reflective, second‐harmonic generation, spatial

## Abstract

In the past decades, metasurfaces have opened up a promising venue for manipulating lights and electromagnetic (EM) waves. In the field of nonlinearity, second‐harmonic generation (SHG) is a research focus due to its diverse applications. There have been many researches for realizing SHG in optical regime using nonlinear characteristics of optical materials, but its efficiency is low. In microwave frequencies, SHGs are basically studied in the guided‐wave systems. Here, high‐efficiency SHGs of spatial waves are presented in the microwave frequency using nonlinear metasurface loaded with active chips at the subwavelength scale. The nonlinear meta‐atom is composed of receiving antenna, transmitting antenna, and active circuit of frequency multiplier, which can realize strongly nonlinear response and link the EM signals from the receiving to transmitting antennas. Correspondingly, to achieve the function of spatial‐wave frequency multiplication, the working frequency of the transmitting antenna in the meta‐atom should be twice as that of the receiving antenna, and hence the active chip is well matched to obtain the signal transforming with high efficiency. Good performance of the spatial‐wave frequency multiplication is demonstrated in the proof‐of‐concept experiments with the best transform efficiency of 85.11% under normal incidence, validating the proposed method.

## Graphical abstract

A strongly nonlinear metasurface loaded with active circuits of the frequency multiplier at subwavelength scale is proposed. Composed of receiving antennas, transmitting antennas, and active circuits of frequency multiplier, the metasurface can realize spatial‐wave frequency multiplication with high efficiency in the microwave frequency. The frequency bandwidth of the metasurface can be adjusted by modifying the geometrical parameters.

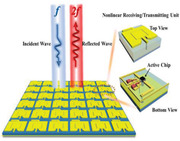

## 1 Introduction

Optical nonlinearities have been observed in natural media when interacting with light at high intensities, and such nonlinear microscopic properties are intimately related to an effectively macroscopic nonlinear polarization, resulting in power conversions from some frequencies to others.^[^ ^1^, ^2^, ^3^, ^4^ ^]^ Second‐harmonic generation (SHG) is one of the most important nonlinear effects, and has been utilized to diverse applications, such as source generation,^[^ ^5^ ^]^ communication,^[^ ^6^ ^]^ imaging,^[^ ^7^ ^]^ and vital signs monitoring.^[^ ^8^, ^9^ ^]^ In the optical frequency, SHG is a nonlinear wave‐mixing procedure where two same‐fundamental‐wavelength (*λ*_FW_) incident photons acting together to generate a single photon with higher energy at the second harmonic wavelength (*λ*_SH_ = *λ*_FW_/2).^[^ ^10^ ^]^ Traditionally, SHG can be desinged using a noncentrosymmetric crystal superlattice of bulk material with large nonlinear susceptibility,^[^ ^11^ ^]^ and more importantly, accurate phase matching is required for efficient frequency conversions in the conventional nonlinear‐optical crystals.^[^ ^12^ ^]^

The emergence of metamaterials and metasurfaces have opened up a promising venue for artificially manipulating the electromagnetic (EM) waves in the linear optical area at subwavelength scale.^[^ ^13^, ^14^, ^15^ ^]^ Recently, investigations of optical metamaterials and metasurfacs exhibit some novel nonlinear properties and can relax the critical phase matching requirement, inlcuding phase‐mismatch‐free nonlinear generation,^[^ ^16^ ^]^ large nonlinear susceptibilities,^[^ ^17^, ^18^, ^19^ ^]^ and new quasiphase matching capabilities,^[^ ^17^, ^18^ ^]^ which were not found in natural nonlinear buck optical crystals.^[^ ^20^, ^21^ ^]^ Therefore various optical metasurfaces have been proposed for enhancing SHG, and the intensity, phase, and polarization states of the induced SHG can be precisely manipulated by optimizing the sizes, shapes, and orientations of the metallic nanostructures.^[^ ^10^, ^22^, ^23^, ^24^, ^25^, ^26^, ^27^, ^28^, ^29^, ^30^ ^]^

The traditional optical buck metamaterial can generate second harmonic nonlinearity with efficient conversion by using phase matching techniques. The optical metasurfaces have exhibited novel nonlinear properties that were not found in the natural nonlinear buck optical crystals, and they provide a promising venue for enhancing and manipulate SHGs. However, in the microwave band, the above mentioned nonlinear properties are not found in natural materials.^[^ ^31^ ^]^ Actually, the passive meta‐structures cannot contribute to the nonlinear design, and the passive resonance features of the metasurfaces cannot be directly employed for the generation of nonlinearity in the microwave frequency. After the composite materials created by arrays of wires and split‐ring resonators (SRRs), also referred as left‐handed materials, were demonstrated to have property of negative refraction, researchers have theoretically and experimentally shown that left‐hand materials constructed with varactor‐loaded SRRs can exhibit dynamic tunability and self‐induced nonlinearity.^[^ ^32^, ^33^, ^34^, ^35^, ^36^, ^37^ ^]^ Therefore, in the past decade, the nonlinear metamaterials with active devices have been extensively investigated in the microwave frequencies.^[^ ^38^, ^39^, ^40^, ^41^, ^42^, ^43^ ^]^ For instance, by inserting a varactor diode into the gap of SRR, the SHG intensity can be enhanced in quasiphase‐matching configurations and double‐resonant particles.^[^ ^17^, ^38^ ^]^ However, these works have been analyzed to generate SHGs at low powers with low efficiency.^[^ ^39^, ^40^ ^]^ Some plasmonic metamaterials with compact size and efficiency have been proposed.^[^ ^41^, ^42^, ^43^ ^]^ The ultrathin spoof surface plasmon polaration meta‐waveguides loaded with a field effect transistor (FET)^[^ ^41^ ^]^ and varactor diodes^[^ ^42^, ^43^ ^]^ can realize efficient SHGs. More importantly, these SHG works are limited to the guided waves.

Recently, owing to the simultaneous manipulations of the EM waves in both space and frequency domains, the space‐time‐coding digital metasurfaces^[^ ^44^, ^45^, ^46^, ^47^, ^48^ ^]^ and time‐domain digital coding metasurfaces^[^ ^49^, ^50^, ^51^ ^]^ have attracted growing interests and enabled efficient harmonics wave‐manipulation capabilities in the microwave frequencies. However, the spatial‐wave SHGs in the space‐time‐coding digital metasurfaces can only generate the second harmonics of the modulation frequency, instead of the carrier frequency of the incident waves.

Therefore, in the microwave band, it is possible to produce spatial‐wave SHGs by integrating microwave active chips with dedicated design of meta‐atom. In this paper, we present high‐efficiency SHGs of the spatial waves and realize spatial‐wave frequency multiplication in the microwave frequency using strongly nonlinear metasurfaces loaded with active circuits of frequency multiplier at subwavelength scale. The nonlinear meta‐atom is composed of a receiving antenna, a transmitting antenna, and an active chip. To achieve the second‐harmonic generator, the operating frequency of transmitting antenna in the nonlinear metausurface should be designed as twice as that of receiving antenna. In addition, to make the EM signals transform with high efficiency, the active chip of frequency multiplier needs to be well matched with the transmitting and receiving antennas. According to the simulations and experimental measurements, the results demonstrate efficient frequency multiplications of incident spatial waves by the proposed nonlinear metasurface.

## 2 Results and Discussion

### 2.1 Design of Strongly Nonlinear Metasurface

**Figure 1:**
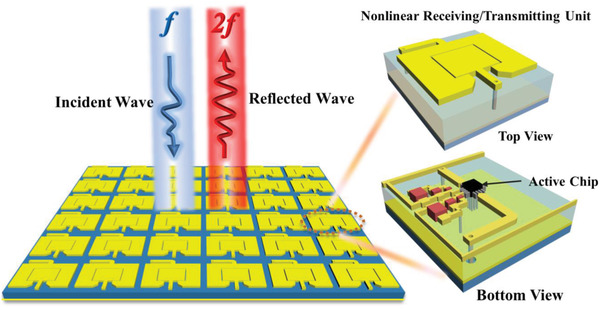
SHG by strongly nonlinear metasurface. The nonlinear metasurface is composed of a 2D lattice of subwavelength‐scale meta‐atoms. Each meta‐atom is consists of a receiving antenna, a transmitting antenna and an active chip. The active chip is forming an active circuit of microwave frequency multiplier embedded on the bottom of the meta‐atom, which can be considered as a bridge to link the electromagnetic signals from the receiving antenna to the transmitting antenna. The 3D structure of nonlinear metasurface unit is presented in the zoom‐in inset of this figure. When the spatial incidence captured by the receiving antenna goes through the integrated nonlinear active chip, the second harmonic signal can be generated and radiated back again into space via the transmitting antenna.

**Figure 2:**
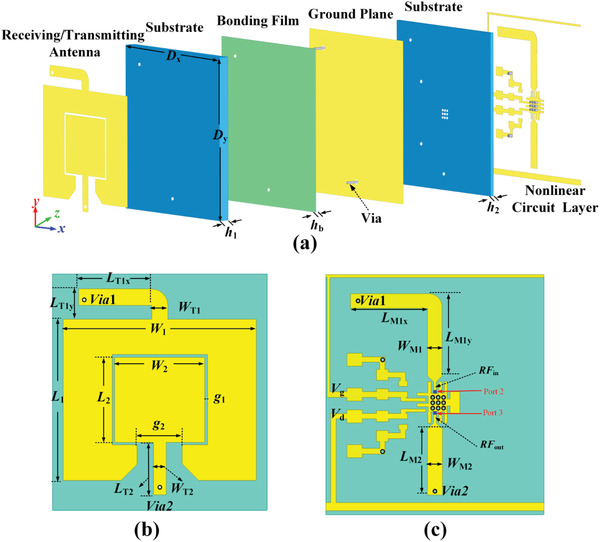
Topological structure of the proposed second‐harmonic meta‐atom. a) Explosive view of the multilayer structure. b) Top view of the receiving and transmitting antenna. c) Top view of the nonlinear circuit layer.

**Figure** **1** illustrates a schematic diagram of SHG using nonlinear metasurface, which is composed of 2D lattices of subwavelength‐scale meta‐atoms. Each meta‐atom is composed of a receiving antenna, a transmitting antenna, and an active chip. The active chip forms an active circuit of microwave frequency multiplier embedded on the bottom of meta‐atom, which can be considered as a bridge to link the EM signals from the receiving antenna to the transmitting antenna. The 3D structure of the meta‐atom is presented in the zoom‐in inset of Figure 1. When the spatial incidence captured by the receiving antenna goes through the integrated nonlinear active chip, the second harmonic signal can be generated and radiated back again into space via the transmitting antenna. The topological structure of the proposed meta‐atom unit is shown in **Figure** **2**a. It is a three‐copper‐layer printed circuit board structures supported by two substrates and one bonding film. The three copper layers are receiving/transmitting antenna, ground plane, and nonlinear circuit layer, respectively. The two substrates are designed by F4B with dielectric constant (*ε*_r_ = 2.65) and loss tangent (tan*δ* = 0.002). The thicknesses of two substrates are 1.5 and 0.5 mm, respectively. The bonding film is designed with a dielectric constant of 3.52 and thickness of 0.1 mm.

The receiving/transmitting antenna is composed of two patches. The rectangular inner patch is the receiving antenna and is used for receiving fundamental‐frequency (*f*_FF_) incident wave. The outer patch with an open loop fed at reversal direction is the transmitting antenna, which can be used for radiating second‐harmonic‐frequency (*f*_SH_) reflected wave with the same polarization. The two patches are connected with the bottom layer by two *λ*/4‐transformers and two metal via‐holes through the ground. The components in the bottom layer contain an active chip of frequency multiplier and a related peripheral circuit. Besides, the electronic circuit shares the same ground with the receiving/transmitting antenna, and ground signals in the active circuit can be directly connected to ground plane by metalized via‐holes. Therefore, the second harmonics generation and transmitting route can be demonstrated by the topological structure clearly. The spatial EM wave of frequency *f*_FF_ is captured by the front receiving antenna and converted to the radio frequency (RF) signal. Then the RF signal transmits through one via‐hole to the bottom nonlinear circuit layer and is converted to the RF signal of second‐harmonic frequency *f*_SH_ by the active circuit of frequency multiplier. Finally, the output RF signal of *f*_SH_ is sent to the front transmitting antenna through another via‐hole and is transformed into the reflective spatial EM wave radiating back again into space.

To realize the second‐harmonic generator, the operating frequency *f*_SH_ of transmitting antenna in the nonlinear metasurface should be designed as twice as the *f*_FF_ of receiving antenna. The top view of receiving and transmitting antenna is shown in Figure 2b. For frequency *f*_FF_, the receiving antenna works in a higher‐order mode, and the resonance is associated not only with the physical length in *y*‐direction, but also with the length in *x*‐direction. For frequency *f*_SH_, the transmitting antenna operates in the basic‐order mode, and the response is mainly dependent on its physical *y*‐direction length. Therefore, the key geometrical parameters of receiving/transmitting antenna are physical length in *y*‐direction and can be first estimated by the empirical formula using effective dielectric constant under the certain condition of substrate thickness, receiving patch width and transmitting patch width. The *λ*_ei_ is the corresponding propagation wavelength in the equivalent medium for EM wave of frequency. In addition, two patches are matched to 50 Ω using *λ*_ei_ /4‐transformers for impedance matching of operating point, and *λ_e_*_1_ /4‐transformer for the frequency *f*_FF_ is bent to 90° to minimize the unit size (as shown in Figure 2b). Simultaneously, in order to ensure good impedance matching, the geometries of the bottom nonlinear circuit layer are also required to be optimized carefully, as illustrated in Figure 2c. Furthermore, the smooth conversion parts are also added between the microstrip and the input/output pads of the active chip to obtain the impedance matching between the connecting microstrip and the active chip. Finally, to implement the foregoing concept, the detailed meta‐atom is designed at fundamental frequency (*f*_FF_ = 5 GHz) and second‐harmonic frequency (*f*_SH_ = 10 GHz). We optimize the geometries of the meta‐atom unit so as to cover the operating bandwidth of the nonlinear active chip. The detailed dimension parameters of the proposed nonlinear meta‐atom are provided in **Table** **1**.

**Table 1:** Detailed dimension parameters of the proposed nonlinear receiving/transmitting unit in millimeters

Parameter	*h*_1_	*h*_2_	*h*_b_	*D*_x_	*D*_y_	*L*_1_	*W*_1_	*g*_1_
Value	1.5	0.5	0.1	20	22	15.5	18	0.3
Parameter	*L*_2_	*W*_2_	*g*_2_	*L*_T1x_	*L*_T1y_	*W*_T1_	*L*_T2_	*W*_T2_
Value	8.1	8.4	4.3	6.6	3.0	1.6	4.9	1.3
Parameter	*L*_M1x_	*L*_M1y_	*W*_M1_	*L*_M2_	*W*_M2_			
Value	7.05	7.55	1.4	6.08	1.4			

**Figure 3:**
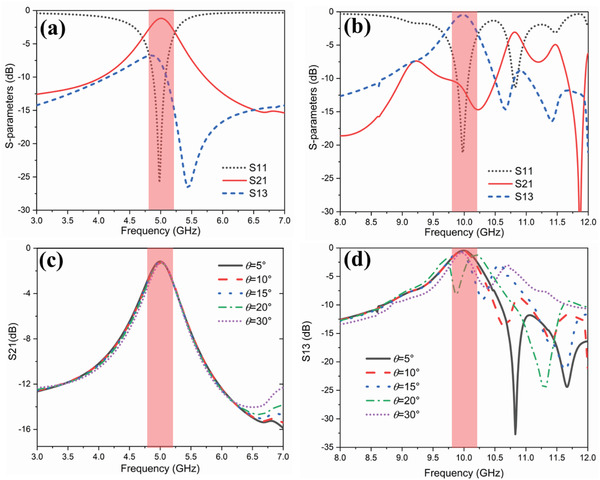
The simulated results of the scattering *S* parameters of the second‐harmonic meta‐atom structure without the nonlinear active chip, in which the input (*RF*_in_) and output (*RF*_out_) of the frequency multiplier are replaced by Ports 2 and 3, and the Floquet Port 1 (with the oblique incidence *θ*) is defined as the transmitting or receiving port of the spatial wave. a) Resonant features at 5 GHz (the fundamental frequency) with the oblique incidence of 10°. b) Resonant features at 10 GHz (the second‐harmonic frequency) with the oblique incidence of 10°. c,d) S21 and S13 simulation results with different oblique incidences of *θ *= 5°, 10°, 15°, 20°, and 30°, respectively.

To demonstrate our design, we perform full‐wave simulations using commercial EM software ANSYS HFSS 2018. The element simulation applies periodic boundary conditions and Floquet port excitation. The input (*RF*_in_) and output (*RF*_out_) of the frequency multiplier are replaced by Port 2 and Port 3 (marked in Figure 2c), and the floquet Port 1 (oblique incidence of 10°) is defined as the transmitting and receiving port of the spatial wave. **Figure** **3** shows the simulated scattering *S* parameters for the proposed whole structure without the active circuit of frequency multiplier. The reflection coefficient S11 and transmission coefficient S21 results are illustrated in Figure 3a. It is obviously seen that the bandwidth of S21 is 0.42 GHz (8.4%) at −3 dB, covering from 4.79 to 5.21 GHz, and the center frequency is 5 GHz. While for the transmission coefficient S13 result shown in Figure 3b, the bandwidth of S13 is 0.49 GHz (4.9%) at −3 dB, covering from 9.71 to 10.2 GHz, and the center frequency is 9.96 GHz. In addition, the S11 results show that the resonant frequencies in the two bandwidths are 5 and 10 GHz with very low reflection coefficient, respectively. Therefore, the simulation results indicate that this meta‐atom has the largest receiving efficiency at fundamental frequency. While the structure has the greatest radiating efficiency at second‐harmonic frequency, which can transfer the most generated second‐harmonic energy into the space through the transmitting antenna.

**Figure 4:**
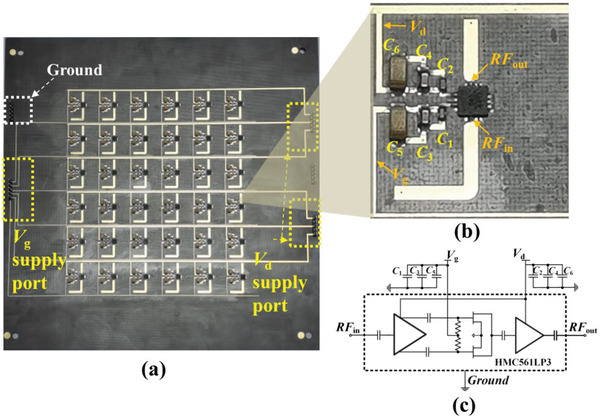
Nonlinear circuits design in the metasurface. a) Photograph of the fabricated metasurface sample (bottom view) composed of 6 × 6 meta‐atoms with the size of 200 × 200 mm^2^ including biasing networks. b) Zoom‐in of the bottom view of nonlinear metasurafce unit sample. c) Function diagram of the active chip frequency multiplier and the related application circuit of active frequency multiplier.

It is well known that designing a high‐quality frequency multiplier is a challenging task which requires nonlinear analysis, matching at the multiplier operating point, stability analysis, and heat dissipation considerations.^[^ ^52^ ^]^ Compared with diode‐based frequency multiplier, transistor‐based, especially FET, can provide better bandwidth with lower input power, DC power, and higher conversion efficiency (or gain).^[^ ^52^ ^]^ Therefore, a FET‐based x2 active frequency multiplier (ADI HMC561LP3) is employed to integrate in our meta‐atom. It is a commercially GaAs monolithic microwave integrated circuit (MMIC) with a broadband input frequency range from 4 to 10.5 GHz. The related application circuit of active frequency multiplier is provided in **Figure** **4**. To guarantee pure DC power supply, the values of decoupling capacitors (*C*_1_–*C*_6_) for the active chip are 100 pF, 100 pF, 1 nF, 1nF, 2.2 µF, and 2.2 µF, respectively. The bottom view of nonlinear metasurface unit sample is provided in Figure 4b.

According to the function diagram of the active chip illustrated in Figure 4c, the key elements are two balanced FETs. The output characteristics of two balanced FETs are determined and tuned by the DC bias voltage *V*_g_, which is connected to the gates of FETs. After the electromagnetic signal goes through the input of chip, there are two complementary signals with 180° phase difference output from the preamplifier and fed as amplified input signal to the common gates. When the bias voltage *V*_g_ is with the range from −2 to −1.2 V, which is higher than the turn‐on‐voltage of FET, then the FET can be turned on and the obtained drain current has rich harmonic components. Under the precondition of the *V*_g_ keeping the same in the work process, the final output of the chip can be calculated as follows

(1) $$\begin{array}{ccc} V_{\mathrm{SH}} & = & A_{\mathrm{post}}\left(f_{\mathrm{SH}}\right)\left[\mathrm{func}\left(V_{g}+A_{\mathrm{pre}}\left(f_{\mathrm{FF}}\right)V_{\mathrm{in}}\right)\right.\\ & & \left.+\;\mathrm{func}\left(V_{g}-A_{\mathrm{pre}}\left(f_{\mathrm{FF}}\right)V_{\mathrm{in}}\right)\right] \end{array}$$

in which *V*_in_ means the input signal voltage of electromagnetic signal and func(•) represents the voltage response function of FET. *A*_pre_(*f*_FF_) and *A*_post_(*f*_SH_) are the transmission‐spectrum functions describing the linear response of preamplifier and postamplifier in the chip, respectively. Therefore, according to Ref. [52], the conversion efficiency can be obtained with the follow equation.

(2) $$\begin{array}{ccc} \eta_{c} & = & \frac{P_{\mathrm{SH}}}{P_{\mathrm{FF}}}=\frac{V_{\mathrm{SH}}^{2}/R_{L}}{V_{\mathrm{in}}^{2}/R_{i}}=\frac{R_{i}}{R_{L}}\cdot A_{\mathrm{post}}^{2}\left(f_{\mathrm{SH}}\right)\\ & & \times {\left[\sum_{n=1}^{\infty }\frac{\mathrm{fun}c^{\left(2n\right)}\left(V_{g}A_{\mathrm{pre}}\left(f_{\mathrm{FF}}\right)\right)}{n!}V_{{}_{\mathrm{in}}}^{n-1}\right]}^{2} \end{array}$$

According to Equation (2), the conversion gain of the HMC561LP3 is estimated with 7.94 from 4 to 10.5 GHz, and we can learn that the efficiency is related with input power and DC bias voltage *V*_g_.

### 2.2 Measurement Results and Discussion

**Figure 5:**
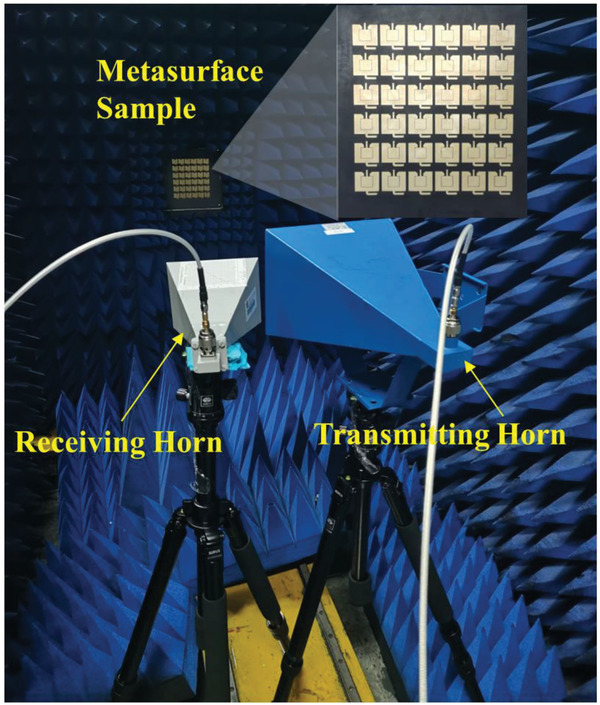
Photograph of the fabricated metasurface sample (top view) and experiment setup for the power spectrum measurements of SHG in the microwave anechoic chamber.

**Figure 6:**
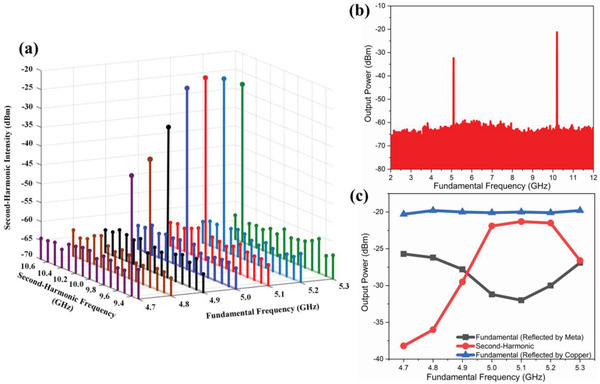
Measured results of the second‐harmonic metasurface generator. a) Frequency‐doubled wave reflecting from the nonlinear active metasurface. The reflective SHG spectra generated with a series of *f*_FF_ incident wave from 4.7 to 5.3 GHz. The input power of incident wave is maintained at a constant level by setting the output intensity of the signal generator as 10 dBm. For each incident excitation wave with the frequency *f*_FF_, there is a distinct peak at the corresponding second‐harmonic frequency 2*f*_FF_ in the reflected spectrum. b) Measured frequency spectra of the second‐harmonic metasurface generator at the excitation frequency of 5.1 GHz. c) Results of the reflected fundamental frequency and SH powers at a series of incident *f*_FF_, compared with the reflected fundamental frequency intensity from the copper.

To validate our proposed concept and design, we fabricate and measure a sample of nonlinear active metasurface composed of 6 × 6 meta‐atoms with same parameters illustrated in Table 1, as shown in **Figure** **5**. The sample is manufactured with the size of 200 × 200 mm^2^. The bottom and top views are shown in Figures 4a and 5, respectively. The biasing networks consist of biasing wires for the DC bias voltage *V*_g_ and the ones for the 5V DC power supply voltage *V*_d_, which can make the active chip work appropriately. All capacitors and the active chip are embedded on the bottom layer of metasurface sample with the use of surface mount machine soldering techniques, as shown in Figure 4b. The experiments are performed in the microwave anechoic chamber, and measurement setup is also provided in Figure 5. Please refer to the Experimental Section for more information about the experimental setup. First, the nonlinear spectrum of the reflective SHG under an oblique incidence of 5° is measured and shown in **Figure** **6**. The incident wave *f*_FF_ varies from 4.7 to 5.3 GHz with a step of 0.1 GHz, and the output intensity of the signal generator for excitation is retained at a constant level of 10 dBm. It can be observed that for each incident wave with frequency *f*_FF_, there is a distinct peak at corresponding second‐harmonic frequency 2*f*_FF_ in the reflected spectrum. The three maximum induced SHG power are identified with −21.3, −21.5, and −21.9 dBm at the excitation frequency of 5.1, 5.2, and 5 GHz. As a typical example, the measured frequency spectrum at *ƒ*_FF_ = 5.1 GHz is shown in Figure 6b. We can obviously observe an output peak power at 10.2 GHz, which is the second‐harmoinc frequency, accompanying with a weaker gain at fundamental frequecny. For the conveinet quantitative analysis, the results of the reflected intensity of fundamental frequency and SHG at broadband incident frequencies are compared with ones from the copper, as illurtated in Figure 6c. The measured bandwidth of efficient SHG has good consistency with simulation. Here, a copper plate with same size is placed in the same position of metasurface sample for the comparison. The measured results suggest that the detected fundamental frequencuy wave intensity reflected from copper keep nearly unchanged with incident wave *f*_FF_ varies, maintaining at a constant level of −20 dBm when setting the signal generator output intensity at 10 dBm.

**Figure 7:**
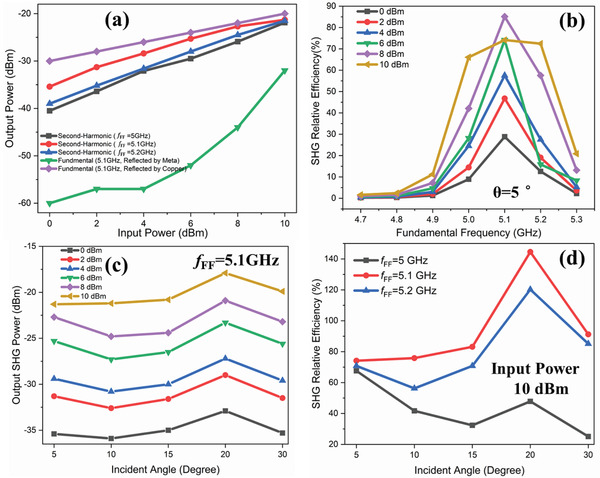
The nonlinearity of SHG in the proposed metasurface. a) Relationship of the measured input and output intensities of the reflected waves at the fundamental frequency and second harmonic from the proposed metasurface sample and copper plate. b) The relative conversion efficiencies of SHGs at a series of frequencies and intensities of the incident fundamental waves. c) The measured output intensities of the reflected second‐harmonic waves from the metasurface sample under different oblique incidences with the excitation frequency of *f*_FF_=5.1 GHz. d) The calculated relative conversion efficiencies of SHGs at different oblique incidences (*θ*=5°, 10°, 15°, 20°, and 30°) with the constant input power of 10 dBm.

Then, the nonlinearity of SHG varing the incident power at the oblique incidenct angle of 5° is meausured and shown in **Figure** **7**a. The output power of the signal generator is adjusted from 0 to 10 dBm with the step of 2 dBm, and the frequency is selected as 5, 5.1, and 5.2 GHz. The red‐circle points depict the measured reflected second‐harmonic intensity at *f*_FF_ = 5.1 GHz. The red solid line is the corresponding fitting curve, representing the relationship between input and output intensity. Compared with the linear relationship between the measured input and output intensity of reflected fundamental frequency wave from copper, the active metasurface sample shows a significant nonlinearity at input frequency of 5.1 GHz. Simultaneously, the weaker nonlinearities are observed at *f*_FF_ = 5 and 5.2 GHz. This is mainly because in the fabricated sample, the structure has the largest receiving and radiating efficiency at 5.1 and 10.2 GHz, and has better matching with frequency multiplier, which has a little frequency offset with the simulations. We can also verify this analysis from the measured results of reflected fundamental frequency wave intensity illustrated in Figure 6c, in which a minimum fundamental power reflected from metasurface is identified at *f*_FF_ = 5.1 GHz.

Finally, the quantitative evaluation is carried out. Owing to the conversion efficiency of spatial SHG produced by proposed metasurface cannot be directly calculated by traditional equation based on the above experimental setup in Figure 5, a relative efficiency is provided for estimating the overall conversion efficiency

(3) $$\eta_{r}=\frac{P_{\mathrm{SHR}}}{P_{\mathrm{FFR}}}\times 100\%$$

in which *P*_SHR_ reflects the power of second‐harmonic obtained by the receiving horn, while *P*_FFR_ represents the power of received fundamental frequency reflected from copper under the condition of same size and input excitation power. Thus, when the incident frequency varies from 4.7 to 5.3 GHz with a step of 0.1 GHz, and the excitation power varies from 0 to 10 dBm with a step of 2 dBm, then the relative efficiency of the SHG is measured and calculated, as shown in Figure 7b. It can be observed that a peak value (pink diamond point) of *η*_r_ = 85.11% at *ƒ*_FF_ = 5.1 GHz with input intensity of 8 dBm, where the reflected power of waves are *P*_SHR_ = −22.7 dBm and *P*_FFR_ = −22 dBm, respectively. It means that a reflective spatial second‐harmonic generator with high efficiency and gain can be implemented using our proposed nonlinear active metasurface. In addition, according to the curves at *ƒ*_FF_ = 5.1 GHz with input intensity of 10 dBm in Figure 7, the output SHG power increases, while the efficiency decreases compared with input power of 8 dBm. This is owing to the input characteristics of used active chip in each meta‐atom, whose maximum input‐driven power is 6 dBm. Therefore, when the input power exceeds this threshold, it will cause saturation in the output power of SHG.

The conversion performance under different oblique incidences has been measured (see the Supporting Information S1). Figure S1 (Supporting Information) demonstrates the photograph of power spectrum measurements of SHGs under different oblique incidences (*θ* = 10°, 15°, 20°, 30°) in the microwave anechoic chamber. The measured output intensities of the reflected waves at the fundamental frequency and second harmonic from the metasurface sample and copper plate at different oblique incident angles are shown in Figure 7c; and Figure S2a–c (see the Supporting Information S1). We observe that the output intensities of the reflected second‐harmonic waves keep nearly stable when the incident angle varies except at *θ* = 20°. Figure 7d illustrates the calculated relative conversion efficiencies of SHGs under different oblique incidences. The red circle points represent the conversion efficiencies at *ƒ*_FF_ = 5.1 GHz with the input intensity of 10 dBm, where *η*_r_ = 74.13%, 75.85%, 83.17%, 144.54%, and 91.20% for *θ* = 5°, 10°, 15°, 20°, and 30°, respectively. The conversion efficiencies at *ƒ*_FF_ = 5.2 GHz also behaves similar performance with the variation of incident angle. The transform efficiencies under different oblique incidences with the input powers from 0 to 8 dBm are shown in Figure S2d–h. We observe that the proposed metasurface can achieve the relative conversion efficiencies above 60% at different oblique incidences under the conditions of input excitation powers 8 and 10 dBm with the fundamental frequencies 5.1 and 5.2 GHz. Hence the same meta‐atom can achieve efficient SHG conversions at multiple incident angles.

We remark that the transform efficiency has significant changes at the incident angle of 20°, as shown in Figure 7d, in which *η*_r_ = 144.54% (red circle points) at *ƒ*_FF_ = 5.1 GHz and *η*_r_ = 120.22% (blue triangle points) at *ƒ*_FF_ = 5.2 GHz. In order to investigate the reason for the significant change, numerical simulations are carried out. Figure 3c,d illustrates S21 and S13 results under different oblique incidences, respectively. From Figure 3c, we notice that the power transmissions from the incident wave to the input pin (*RF*_in_) of the active chip (S21) keep nearly the same under different incident angles. However, according to the simulation results of S13 in Figure 3d, a frequency shift (the green dashed dots) occurs in the power transmissions frequency from the active chip output pin (*RF*_out_) to the free space at *θ* = 20°, which is consistent with the measurement result. Therefore, the transform efficiencies have significant changes at the incident frequencies of 5.1 and 5.2 GHz at the incident angle of 20°.

In the current experiments, a DC source is used to connect our designed metasurface sample during the measurements. In the future study, it can be replaced by the onboard power supply circuit module to make the metasurface more integrated and modular. Simultaneously, how to reduce the total cost should also be considered to enhance the application potentials and extend the application scope.

## 3 Conclusion

We proposes a strongly nonlinear metasurface to realize the frequency multiplication of the spatial waves at the microwave frequencies. By integrating the active circuit of frequency multiplier as the bridge to link the receiving antenna and transmitting antenna and making perfect impedance matching with the active chip, a high conversion efficiency of the second harmonic is achieved. A metasurface sample with 6 × 6 meta‐atoms is designed, fabricated, and measured. The experimental results have good agreements with numerical simulations. For the input fundamental frequency of 5.1 GHz, a maximum relative conversion efficiency of 85.11% is realized under normal incidence, which demonstrates a great nonlinear performance of our design. We also show that high conversion efficiencies of SHGs can be achieved under different oblique incidences. It is expected that the proposed metasurface has latent abilities in spatial spectrum shifts, new EM stealth technology, and advanced radar and communication systems.

## 4 Experimental Section

The setup for measuring the power spectrum of SHG is shown in Figure 5. To measure the reflective spectral power distributions, two linearly polarized (LP) horn antennas were mounted on two supporting tripods. The right rectangular horn antenna (blue horn in Figure 5) working from 4.6 to 7 GHz was alternated as transmitting horn and was connected to a microwave signal generator (Agilent E8257D). While the left rectangular horn antenna (white horn) working from 8 to 12 GHz was named as receiving horn and was connected to a spectrum analyzer (Agilent E4447A). The microwave signal generator and spectrum analyzer are not shown in the picture. In order to accurately measure the reflective spectrum, the two horns were all placed as close as possible and 1.5 m away from the metasurface sample. Two DC power sources were utilized to provide voltage sources *V*_g_ and *V*_d_ for the frequency multipliers in the metasurface. The *V*_g_ for chip biasing was set as −1.4 V, and circuits power supply *V*_d_ was set as 5 V. With the aid of the setup, fundamental frequency microwaves were generated ranging from 0 to 10 dBm by using signal generator and transmitting LP horn, while the receiving LP horn received the electric fields of second‐harmonc generated by the metasurface sample and fed into the spectrum analyzer. Therefore, the reflective SHG performances of proposed nonlinear active metasurface that changed with the frequency and intensity of fundamental microwave were tested. In addition, a copper plate with same size is placed in the same position of metasurface sample for the comparison, and the receiving antenna is replaced by a linearly polarized horn antenna working from 4.6 to 7 GHz.

## Conflict of Interest

The authors declare no conflict of interest.

## Supporting information

Supporting Information

## Data Availability

Research data are not shared.
