# Supplementary material for: High‐Efficiency Spatial‐Wave Frequency Multiplication Using Strongly Nonlinear Metasurface
Source: Adv Sci (Weinh). 2021 Jul 15;8(18):2101212. doi: 10.1002/advs.202101212 (PMC8456279; doi:10.1002/advs.202101212)
Supplement: Supplementary file 1 — Supporting Information [file ADVS-8-2101212-s001.pdf]

© 2021 Wiley-VCH GmbH

ADVANCED SCIENCE
Open Access

# Supporting Information

for *Adv. Sci.*, DOI: 10.1002/advs.202101212

## High-efficiency spatial-wave frequency multiplication using strongly nonlinear metasurface

Hai Peng Wang#, Yun Bo Li#*, Shi Yu Wang, Jia Lin Shen, He Li, Shi Jin*, and Tie Jun Cui

Supporting Information

# High-efficiency spatial-wave frequency multiplication using strongly nonlinear metasurface

*Hai Peng Wang#, Yun Bo Li#*, Shi Yu Wang, Jia Lin Shen, He Li, Shi Jin*, and Tie Jun Cui*

## S1. Detailed information of SHG measurements in the proposed metasurface at different oblique incidences

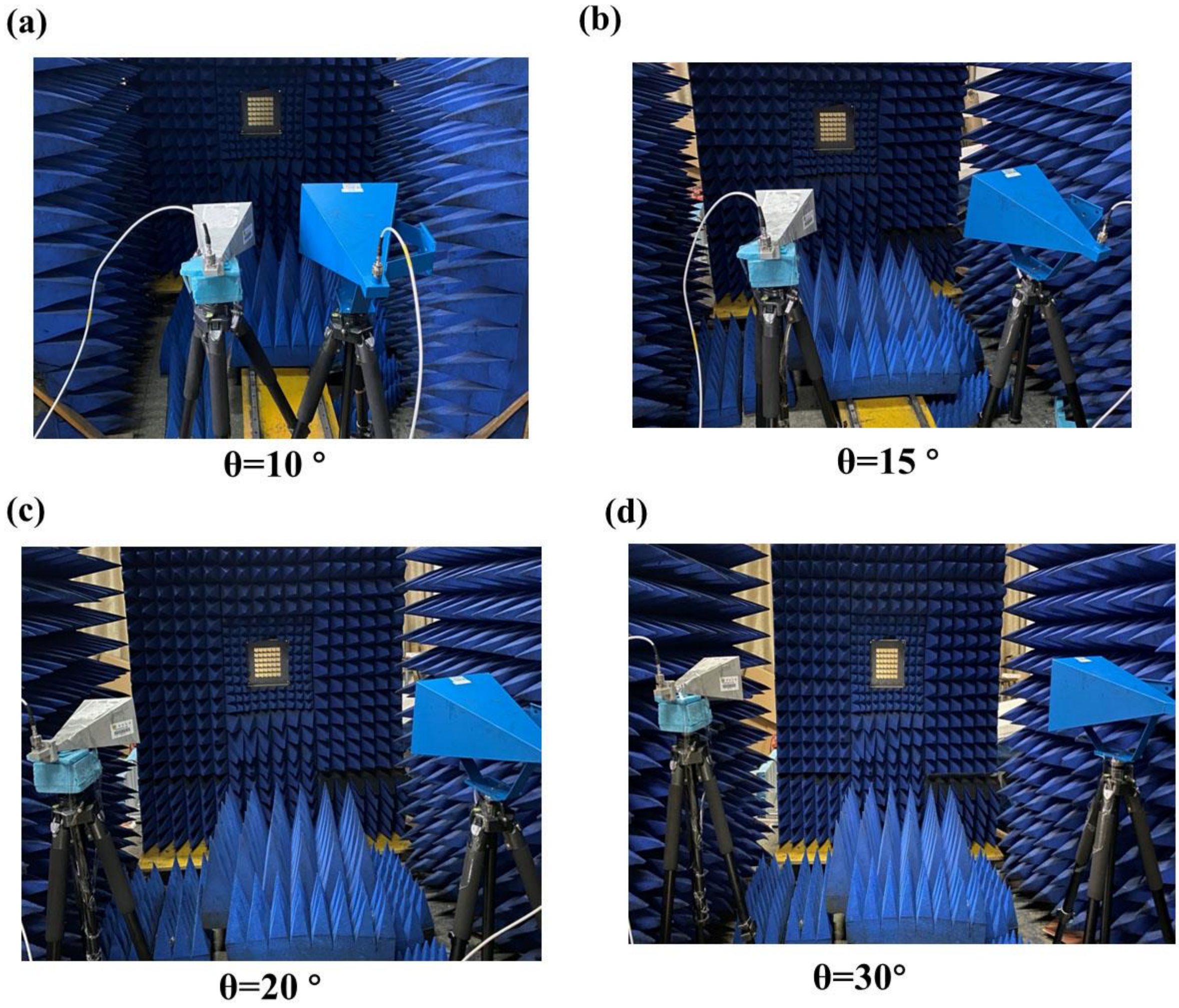


**Figure S1**. The power spectrum measurement setup of SHGs using the proposed metasurface sample under different oblique incidences in the microwave anechoic chamber.

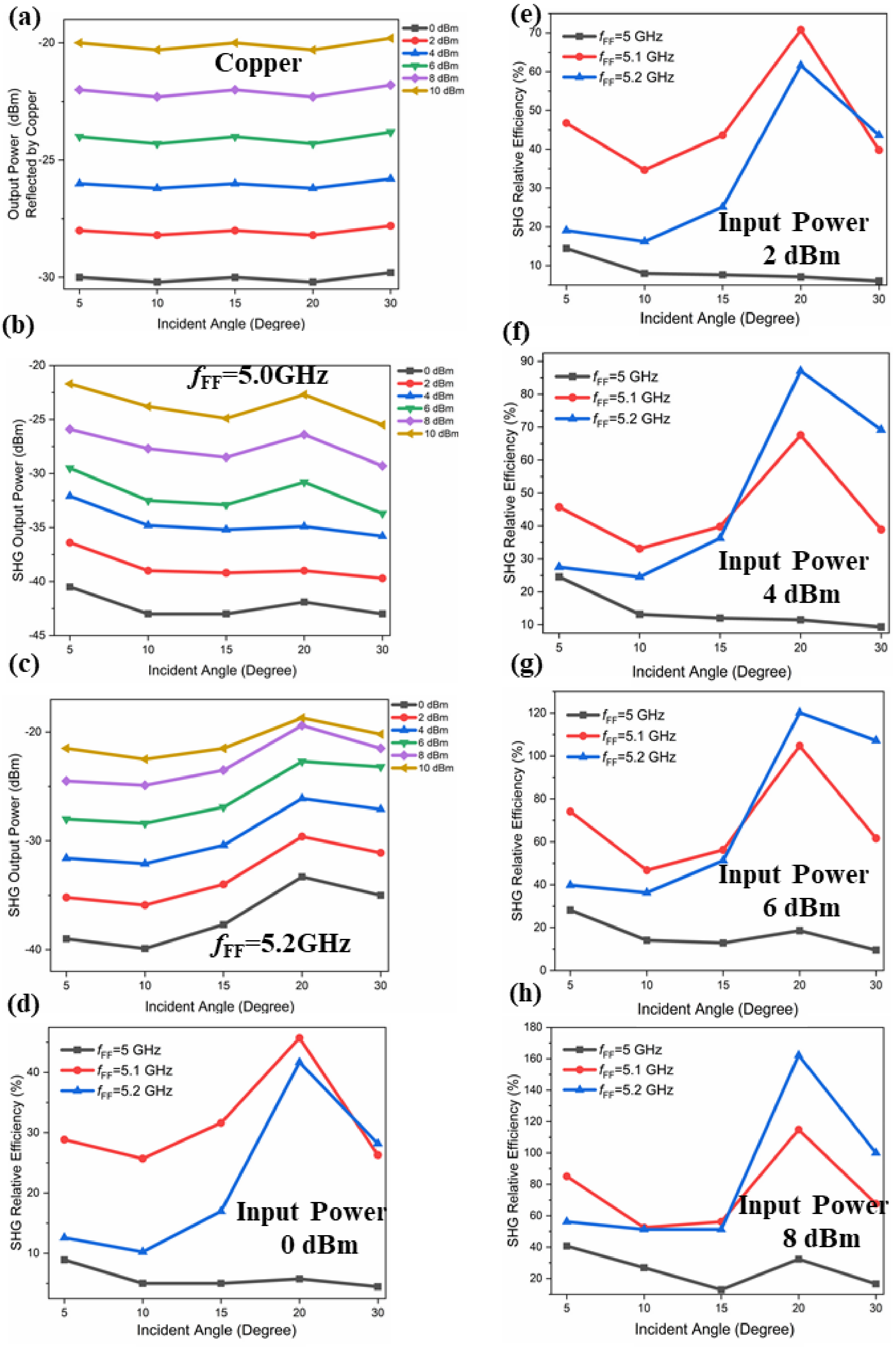


**Figure S2**. (a) The measured output intensities of the reflected waves from the copper plate at different oblique incidences at the frequency of $f_{FF}$=5.1 GHz. (b, c) The reflected second-harmonic waves from the proposed metasurface sample at different oblique incidences with $f_{FF}$=5.0 GHz and 5.2 GHz, respectively. (d)-(h) The calculated relative conversion efficiencies of SHGs under different oblique incidences ($\theta$=5°, 10°, 15°, 20°, and 30°) with the input power varying from 0 to 8 dBm.
